# Rubbery Figures?

**DOI:** 10.1371/journal.pmed.0030070

**Published:** 2006-01-31

**Authors:** Michael Glass

**Affiliations:** **1**Retired teacherNew South WalesAustralia

I have been following reports of the Auvert et al. study [ 1] and have found four different figures for seroconversions.

An abstract from the 3rd International AIDS Conference (Rio de Janeiro, 24–27 July 2005) reported 15 seroconversions in the circumcised group and 45 seroconversions in the uncircumcised group [ 2]. On 29 July 2005, the Science and Development Network reported 18 seroconversions in the circumcised group and 51 in the uncircumcised group [ 3]. A paper in the *New Scientist*, published on 6 August 2005, reported 15 seroconversions in the circumcised group but 51 in the uncircumcised group [ 4]. Finally, on 23 October 2005, a paper in *PLoS Medicine* reported that there were 20 seroconversions in the circumcised group and 49 in the uncircumcised group [ 1]. It seems strange that the figures should have so much variance.

If we just look at the official figures—15 to 45 at the International AIDS Conference and 20 to 49 in *PLoS Medicine*—between 1 August 2005 and 23 October 2005, it appears that there have been four seroconversions among the uncircumcised and five seroconversions among the circumcised. In less than three months, a 3:1 difference has shrunk to a 2.45:1 difference.

Why are the numbers of seroconversions so much at variance in reports published by reputable journals?
